# Masking noise reduces the anti-predator-like response to an acoustic stimulus: Application of Signal Detection Theory to fish behaviour

**DOI:** 10.1371/journal.pone.0327092

**Published:** 2025-07-11

**Authors:** Helen A. L. Currie, Paul R. White, Timothy G. Leighton, Paul S. Kemp

**Affiliations:** 1 International Centre for Ecohydraulics Research (ICER), University of Southampton, Boldrewood Innovation Campus, Southampton, United Kingdom; 2 Institute of Sound and Vibration Research, University of Southampton, Highfield, Southampton, United Kingdom; University of Windsor, CANADA

## Abstract

In studies of animal cognition, the influence of background masking noise on responses to any particular stimulus are often overlooked. In fish, there is little understanding of their response to targeted acoustic stimuli in the presence of high intensity (Sound Pressure Levels) environmental masking noise commonly experienced in the wild. In a controlled laboratory study, Signal Detection Theory was used to investigate coarse (startles) and fine-scale (swimming speed, group cohesion and alignment) responses of common carp (*Cyprinus carpio*) to pulsed tonal signals (170 Hz) differing in their signal-to-noise ratio (low, intermediate, or high) above either background ambient, or masking noise (fixed intensity Gaussian white noise: 120–3000 Hz). In comparison to independent control groups, fish exhibited a startle response, reduced their average swimming speed, increased group cohesion, and became more aligned at the onset of tonal stimuli under ambient noise. Signal discriminability was reduced under the masking noise conditions, with coarse-scale behavioural responses largely absent, and fine-scale responses suppressed but positively related to signal-to-noise ratio. This study enhances understanding of the potential ecological consequences of anthropogenically generated noise on the behaviour of fish and may help in the development of more effective environmental impact mitigation technologies, such as behavioural guidance systems, that use sound to induce avoidance.

## Introduction

Animals obtain vital information from the sounds transmitted between individuals (e.g., birds [1], anurans [2], and insects [3]) and those that emanate from abiotic features within their environment (e.g., flowing rivers [4], wind and rain [5]). Varying over space and time, patterns of acoustic stimuli are responsible for the formation of local soundscapes [6,7], and the signals themselves may be encoded with data concerning landscape structure [8], and population or community composition [9]. Detection of acoustic signals may elicit a contextually dependent behavioural response [10,11], e.g., in relation to habitat selection [12], communication with conspecifics (sexual selection [13], or competition [14]), social aggregation [15], or predator-prey interaction [16]. For some taxa, the value of sound becomes even more critical when other sensory systems are impaired, such as in deep, dark, and potentially turbid aquatic environments in which visual cues may be lacking. Under these conditions, many aquatic organisms are reliant on underwater sound to sense the extended environment and any constraints to receiving acoustically relevant signals could have adverse ecological consequences [17–20].

Global urbanisation (e.g., infrastructure development and transportation) has contributed to an increase in anthropogenic noise worldwide, with negative impacts on aquatic organisms widely recognised (e.g., marine mammals and fish [21], benthic invertebrates [22]). In the case of freshwater fish, exposure to anthropogenic noise can induce negative physiological responses (e.g*.,* increased cortisol levels in blacktail shiner, *Cyprinella venusta* [23]) that are accompanied by modification of the behaviour of individuals and structural dynamics of groups (*e.g*., Eurasian minnow, *Phoxinus phoxinus* [24–26]). High intensity (Sound Pressure Levels, SPL) background noise can disrupt the ability of a fish to extract important biological information from their local soundscape, such as in relation to courtship behaviour observed in spotted (*Gobiusculus flavescens*) and painted gobies (*Pomatoschistus pictus* [27]), or territoriality in red-mouthed gobies (*Gobius cruentatus* [28]). Impacts on orientation [29], predator avoidance [30], and response to chemical alarm cues [31] have also be reported in longspine cardinalfish (*Apogon doryssa*)*,* juvenile European eel (*Anguilla anguilla*) and fathead minnow (*Pimephales promelas*)*,* respectively. Nevertheless, little information exists on the anti-predator-like responses (i.e., behavioural reactions evoked by the threat of a “potential” predator) of fish to acoustic stimuli in the presence of high intensity background noise. This study investigated the influence of masking noise on the response of a freshwater fish to a low frequency (170 Hz) tonal stimulus.

An ability to detect, discriminate, and respond to biologically relevant sounds is dictated by the signal-to-noise ratio (SNR). When energetic background, or masking noise, is of at least equal intensity to that of a signal, and within a critical frequency range [32] and direction [33], it acts as a constraint to signal transmission [34,35]. These relationships are important components of Signal Detection Theory (SDT) [36,37] that provides a framework to better understand the effects of masking on fish response to environmental stimuli (e.g*.*, hydraulic gradients) [38,39]. From a fisheries management perspective, the benefit of such an approach is two-fold. First, SDT may be applied in an attempt to deliberately mask the effects of unwanted environmental stimuli (e.g*.*, in respect to hydrodynamics [39]). Second, SDT may be used to determine a SNR above a masking noise floor (i.e*.*, baseline) at which a signal induces a desired behavioural response. For example, fish guidance technologies (e.g*.*, acoustic behavioural deterrents) may be employed to reduce the risk of injury and mortality of fish at river infrastructure, such as intakes to hydropower turbines, but high levels of background noise often dominate [40,41] and may constrain acoustic signal transmission and the intended response of the target species [42]. Alternatively, the latter may be relevant to minimise the impact of masking from noise pollution on beneficial acoustic communication signals, such as reproductive fish calls. This study will help improve understanding of how fish respond to acoustic signals in the presence of masking noise and the potential wider ecological consequences.

We investigated how the magnitude of an anti-predator-like response exhibited by a group of five fish (common carp, *Cyprinus carpio*) varied when tonal signals were played at three different SPLs (low, intermediate, and high) under ambient and high intensity background masking noise and how this response changed over time. First, we used both coarse: a) startle response; and fine-scale behavioural metrics: b) median group speed (m s^-1^), c) inter-individual distance (m), and d) alignment (°) to quantify the response under the eight treatments (control in which the signal is absent and tones played at three SPLs under both ambient and masking noise). Second, we investigated how the response metrics varied over time compared to a pre-exposure baseline. Finally, we used SDT to accommodate the predisposition of the experimental population, considering probability of detection and individual behavioural responsiveness, or decision-making, under ambient and masking noise conditions. Despite the extent of classic physiological-level studies quantifying auditory masking thresholds in fish [43–48], the influence of background masking noise on fish behavioural response to targeted acoustic stimuli has been commonly overlooked. Given the influence of signal-to-noise ratios on sound detection and discrimination, we made three predictions. First, compared to ambient, under masking noise: (a) an overall reduction in startles in response to an acoustic stimulus would be observed, and (b) swimming speed [49,50], inter-individual distance [24,26,50], and alignment [24–26] would be more variable. Second, the duration over which behaviours that deviated from the baseline were maintained would be greater under ambient noise compared to masking noise. Finally, signal discriminability would be lower and response bias (i.e., probability of incorrect ‘false alarm’ or ‘miss’) greater under masking compared to ambient noise.

## Materials and methods

### Study species and husbandry

Common carp were selected as the model species because of their well-studied auditory sensitivity [51], and interest from both fish conservation (IUCN red listed [52]) and invasive species control [53–55]. In March 2018, 420 juvenile carp were obtained from a hatchery (DC Freshwater Fish, Surrey, UK) and transported to the University of Southampton’s ICER facilities in oxygenated plastic bags containing water from the source aquaria (100% survival during transportation). Fish were acclimated to housing facility water temperatures over a period of two hours before transferral to one of three indoor holding tanks (1.5 m x 1.0 m x 0.78 m; water depth: 0.68 m; stocking density: 1.21 kg/ m^-3^; mean temperatures ± SE: Tank 1: 9.9 ± 1.5°C; Tank 2: 8.9 ± 0.3°C; Tank 3: 9.1 ± 0.3°C) where they acclimatised for three days prior to the start of the experiments. Water quality was monitored to ensure it remained below thresholds considered suboptimal (NO^3-^: < 50 mg L^-1^; NO^2-^: < 1 mg L^-1^; NH_3_: 0; & pH: < 8.4) and maintained using a submersible aerated pump in combination with partial water exchanges when necessary. Fish were held under a 12:12 h light:dark photoperiod cycle and provisioned daily with commercially available aquarium flaked food until satiation. As juvenile carp are a shoaling species they were tested in groups (five fish) to minimise stress [56]. On completion of each trial, fish were measured (standard length ± SE: 68.3 ± 0.8 mm) and weighed (wet mass ± SE: 9.8 ± 0.3 g). Differences (Kruskal-Wallis rank sum) in wet mass (χ^2^ = 21.9; *d.f.* = 7; *p* < 0.01) and standard length (χ^2^ = 14.9; *d.f.* = 7; *p* < 0.05) were a*p*parent between treatments. However, a *post hoc* Dunn’s test indicated deviations between treatments to be only for larger fish exposed to the masking control treatment. As absolute values, these differences were unlikely to influence results (Table 1). The study was approved by the University of Southampton’s Animal Welfare and Ethical Review Body.

**Table 1:** Ambient and masking treatment parameters (and abbreviations) used in an experiment designed to investigate the response of fish to tonal acoustic stimuli in the presence of a high intensity background masking noise. Note: ‘SPL (RMS)’ is the Root Mean Square sound pressure level.

Treatment	Tone SPL (RMS) (dB re 1 µPa)	Abbreviation	n Trials	Mean fish length ±SE (mm)	Mean fish wet mass ±SE (g)
**Ambient control**	n/a	‘AMB-C’	10	66.2 ± 1.9	8.7 ± 0.8
**Masking control**	n/a	‘MASK-C’	10	74.3 ± 1.2	11.8 ± 0.6
**Ambient low SPL**	110	‘AMB-LOW’	10	70.2 ± 2.5	11.1 ± 1.0
**Ambient intermediate SPL**	121	‘AMB-INT’	10	65.4 ± 2.0	8.5 ± 0.7
**Ambient high SPL**	130	‘AMB-HIGH’	10	66.0 ± 2.3	8.1 ± 0.9
**Masking low SPL**	110	‘MASK-LOW’	10	65.8 ± 2.7	9.8 ± 1.0
**Masking intermediate SPL**	121	‘MASK-INT’	10	69.7 ± 1.9	9.9 ± 0.7
**Masking high SPL**	130	‘MASK-HIGH’	10	68.5 ± 2.2	10.2 ± 0.9

### Experimental area

Experiments were performed within a section (86 cm x 30.8 cm x 30.2 cm) of a still-water acrylic tank (300 cm x 30.8 cm x 30.2 cm), housed inside a decommissioned walk-in cold room, repurposed for noise attenuation. Two fully immersed speakers (Electro-Voice UW-30; maximal output 153 dB re 1 µPa at 1 m for 150 Hz, frequency response 0.1–10 kHz; Lubell Labs, Columbus, OH) were suspended at a fixed point in the middle of the water column, one behind a micro-mesh acoustic baffle at either end of the experimental area, to generate the sound field (see Currie *et al*. [24,25], for experimental arena schematics). Water depth remained constant at 27 cm. Every ten trials, water was replaced to minimise the build-up of metabolic byproducts, food waste and pheromones. Tanks were left to settle overnight and for the water to return to room temperature (mean ± SE: 10.9 ± 0.12°C).

The experimental area was enclosed by a wooden frame covered in plastic blackout material to visually isolate the fish from the observer. Light levels remained constant throughout the trials and a white background was attached to the outside of the experimental area and illuminated from underneath using two PhotoSEL Photography bulbs (pure white full-spectrum flicker free; 85 W, 5000 lumen; SJT Commercial Ltd., UK). This increased contrast of the fish for digital video recordings using a webcam (C920; HD 10809; 30 frames s^-1^; Logitech Pro, Switzerland) mounted above the centre of the experimental area.

### Sound stimuli and acoustic mapping

Sound samples were generated using a custom written MATLAB script (Release 2017a, The Mathworks, Inc., Natick, Massachusetts, United States). The signal was sent through a ProSound 200 power amplifier (50 W, frequency response range approx.: 0.02–20 kHz; London, UK) to the underwater speakers via a DAQ (NI USB-9174; National Instruments, U.K) connected to a laptop computer.

**Fig 1:**
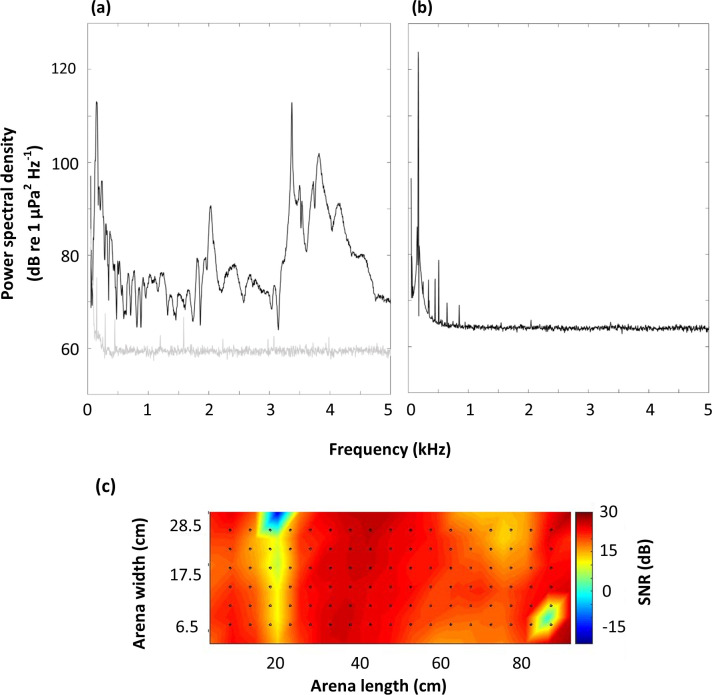
Example power spectral density (dB re 1 µPa^2^ Hz^-1^) of acoustic conditions within the experimental area (sampling rate: 25.6 kHz; FFT size 8192 [3 Hz bin width], overlap 91.5%, von Hann Window, frequency range 50-5000 Hz) for (*a*) baseline ambient noise conditions (bottom grey line) with masking broadband noise (120–3000 Hz) conditions (top black line); and (*b*) 170 Hz tonal stimuli; and (*c*) insonified experimental area showing the dimensionless signal-to-noise ratio (SNR [dB]) in the1/3^rd^ octave band at 13.5 cm depth. Note: dots indicate the location of the hydrophone when measuring the sound field.

Test stimuli of 170 Hz (centred on the 1/3^rd^ octave band: ~ 151–190 Hz) pulsed tones (1 s ON: 2 s OFF) and masking broadband noise of 120–3000 Hz at a fixed intensity of 110 dB re 1 µPa (root mean square: RMS) was used (Fig 1). The selected tonal stimuli was within the known auditory sensitivities of common carp (100–3000 Hz, with lower thresholds observed in the range below 505 Hz [51]). The masking noise was informed by field recordings taken at the Totnes Weir Hydropower plant on the River Dart, Devon (50º26’20.5”N 3º41’23.8”W), on 20 November 2017. Noise samples were recorded for 1-minute at 22 independent points using a hydrophone (Type 8105: manufacturer-calibrated sensitivity −205 dB re: 1V µPa^-1^, frequency response 0.1–160,000 Hz; Brüel & Kjær, Royston, U.K), connected to a charge amplifier (Type: 2635; Brüel & Kjær, Royston, U.K) and audio recorder (model: DR-100MKIII; .wav format, sampling rate 192 kHz; TASCAM, Weisbaden, Germany). Recordings taken from upstream and downstream of the turbine were analysed (dominant frequency range: 0–3 kHz; sound pressure level [SPL], [RMS]: upstream of turbine 118.5 dB re 1µPa; downstream 125.1 dB re 1µPa).

Artificial masking noise stimuli was created by digitally filtering Gaussian white noise, created at a sample rate of 12.8 kHz, and band-passed using an 8^th^ order Butterworth filter. Acoustic intensity of the pulsed tone was played back at either a low (110 dB re 1 µPa), medium (121 dB re 1 µPa), or high (130 dB re 1 µPa) SPL, to create differing signal-to-noise ratios. To avoid lower frequency resonance issues with the underwater speakers during stimuli playback, a high pass filter was applied at 100 Hz. As spatial separation of a sound source can influence the effectiveness of a masker, noise and tonal stimuli were played back through both speakers [57]. Eight acoustic treatments, including an ambient control of no sound (ambient noise: 82 dB re 1 µPa), were used in the experiments (Table 1). In the absence of any acoustic playback during the ambient control, an electrical signal was sent to the speakers to avoid any confounding influences of electroreception. Sound pressure levels of all treatments were standardised in the centre of the experimental area.

Prior to conducting the trials, the acoustic field was mapped to quantify both the tonal stimuli and masking noise, with the latter as broadband noise (120–3000 Hz) in the 1/3^rd^ octave band to reflect the highly frequency selective nature of masking. The 1/3^rd^ octave band approximately represents the smallest band of frequencies that will simultaneously activate the natural auditory filters, causing perception interference to mask the tone (critical band [32]). The hydrophone (Type: 4013: manufacturer-calibrated sensitivity −211 dB re: 1V µPa^-1^, frequency response 0.01–170,000 Hz; Teledyne RESON, Slangerup, Denmark) was connected to a voltage amplifier (Type: A1001; 9 V; gain +40 dB, high pass filter 100 Hz; Etec, Frederiksværk, Denmark) and fixed to a customised rig to measure acoustic intensities at 306 positions within the experimental area. The signal was relayed through the DAQ and back to the laptop computer, from which custom written MATLAB script was used to control and record from the data acquisition system (sampling rate 25.6 kHz; FFT 1024, overlap 50%, Hann window). Hydrophone calibration was confirmed with a pistonphone (type: 4229; Brüel & Kjær, UK). Resulting SPLs were used to describe the sound-field within the experimental area across three different depths (Fig 1; S1 Fig).

The particle acceleration component, *a*, was calculated following Currie *et al.* [24,25], as:


$$a=-\frac{1}{\rho }\nabla P$$ (1)

where *P* is the sound pressure, and *ρ,* the ambient density.

As the sound pressure signal was measured on a regular grid of points (306 positions: 17 x 6 x 3 grid), the pressure gradient could be calculated using a finite difference approach. The root mean square (RMS) of the pressure difference was evaluated independently in the x, y, and z directions and pressure gradient obtained by dividing by the distance between measurements. Using Equation (1), the RMS particle acceleration was calculated in one direction by dividing by the water density. By combining RMS values in all three directions, the total RMS particle acceleration was finally determined. Results were expressed in decibels (dB re 1 µm s^-2^) [58,59] and mapped for the central depth (13.5 cm) of the tank (S2 Fig).

### Experimental protocol

A total of 80 fifty-minute (including 30-minute acclimation time) trials were conducted, comprising ten replicates for each treatment and control. Each replicate consisted of five similar sized naïve carp (n_fish_ = 400), introduced simultaneously to the centre of the experimental area, and each used in one trial only.

For the four masking treatments (‘MASK-C’, ‘MASK-LOW’, ‘MASK-INT’, ‘MASK-HIGH’: Table 1), carp were introduced to the experimental area with the noise playback projecting simultaneously from the two underwater speakers. For the four ambient treatments (‘AMB-C’, ‘AMB-LOW’, ‘AMB-INT’, AMB-HIGH’: Table 1), an electrical signal only was relayed to the speakers. Fish were allowed 30 minutes to acclimate, after which a tonal stimuli was presented for ten minutes (either in combination with or in the absence of masking noise), after which exposure to the stimuli ceased. To avoid order effects, a random number generator (https://www.random.org) was used to determine order of playback.

### Video tracking and behavioural parameters

Video recording commenced on introduction of each group of fish. Videos were played back in a randomly generated order with the single observer blind to treatment. Coarse-scale behaviour was analysed with respect to the exhibition of startle behaviour, confirmed when a fish displayed an escape response at the onset of the tonal stimuli (i.e., within the first second of the ten-minute exposure period), often in terms of a clear burst in swimming at an altered angle compared to the pre-startle speed and trajectory [60]. The number of times at least one individual within a group startled in response to each consecutive pulsed tone without interruption was recorded as the number of “continuous startle responses”. Startles typically lasted for < 0.5 s duration, and periods of continuous startling typically ceased within the first 10–30 s. Videos were reviewed in full-screen mode, with the observer blinded to playback timing, and therefore ignorant to the timing of the tonal stimuli. This was verified only after startle responses were identified. Fine-scale behaviour was investigated by tracking fish movements extracted from video recordings using a custom written MATLAB script (as per Short *et al*. [26]) and quantified (Table 2) in relation to: i) swimming speed (m s^-1^); ii) inter-individual distance (m); and iii) alignment (°), with lower orientation values representing greater alignment [24,25,50,61]. Group swimming speed, inter-individual distance, and alignment were calculated for each frame (30 frames s^-1^), providing an output of 90,000 data points per variable for each fish group.

**Table 2:** Definitions of fine-scale group behaviour of common carp (*Cyprinus carpio*) in response to differing acoustic treatments, quantified from tracked fish movements extracted from video recordings.

Fine-scale behavioural parameters	Definition
**Swimming speed (m s^-1^)**	Mean speed of the ‘mean shoal centre’ of fish within a group relative to other members. The ‘mean shoal centre’ (X_C_(*n*)) [24–26] location of a fish group (in 2D), relative to the walls of a tank, is calculated from the position of the *i*th fish in the *n*th video frame, vector X_*i*_(*n*) = (*x*_*i*_(*n*),*y*_*i*_(*n*))^*t*^ *x*_*i*_(*n*) represents the distance along length of tank, and *y*_*i*_(*n*) the breadth, and therefore: *X*_C_(*n*) = (*x*_*c*_(*n*),*y*_*c*_(*n*))^*t*^=(*X*_*1*_(*n*) + X_2_(*n*) + *X*_*3*_(*n*) + *X*_*4*_(*n*) + *X*_*5*_(*n*))/5
**Inter-individual distance (m)**	Mean distance of the centre point of each fish from the ‘mean shoal centre’ [24–26], quantified by measuring and combining the standard deviations of the locations on the x and y-axis.
**Alignment (°)**	Standard deviation of the angle of the fish (−90° to 90°) compared to one another is calculated using an imaginary horizontal line as a reference through the fish (head to tail), i.e., pointing the same direction or randomly aligned. Lower orientation values represents greater alignment [24–26].

Using the principles of SDT, discriminability (*d’*) and response criterion (*c*) were calculated in relation to observed startles (coarse-scale behaviour) under masked and ambient treatments. As low frequency tones induce changes in inter-individual distance in other cyprinid species (e.g., Eurasian minnow [24,25]), this parameter was used to determine the fine-scale behaviour SDT metrics (Table 3). *d’* is calculated from the hit rate (HR) and false alarm rate (FAR) [38,39] and is measured in standard deviation units (z-scores) for right-tail probabilities of the normal distribution, where:

**Table 3:** Signal Detection Theory: the four potential signal-response outcomes [38] of common carp (*Cyprinus carpio*) to the presence or absence of a tonal stimulus for the coarse and fine-scale behavioural parameters used to quantify SDT metrics.

BEHAVIOUR			RESPONSE	
			Yes	No
**Coarse-scale** Startle response (presence/absence)	**SIGNAL**	**Present**	HIT *i.e., startle response observed in response to tonal signal*	MISS *i.e., no startle response observed despite presence of tonal signal*
		**Absent**	FALSE ALARM *i.e., startle response observed despite absence of tonal signal*	CORRECT NON-RESPONSE *i.e., no startle response observed in absence of tonal signal*
**Fine-scale** Inter-individual distance (m)		**Present**	HIT *i.e., change in inter-individual distance observed in response to tonal signal*	MISS *i.e., no change in inter-individual distance observed despite presence of tonal signal*
		**Absent**	FALSE ALARM *i.e., change in inter-individual distance observed despite absence of tonal signal*	CORRECT NON-RESPONSE *i.e., no change in inter-individual distance observed in absence of tonal signal*


d’ = z(HR) – z(FAR) (2)

Standard corrections were performed on FAR (1/(2N)) when the true p-value was 0 (z= ∞), and for HR (1−1/(2N)) when the true p-value was 1 (z= −∞) [62]. The higher the *d’* value, the higher the level of signal discriminability. *c* assumes an equal probability of incorrect ‘false alarm’ or ‘miss’ [36–38] and is a measure of response bias (Table 3). At value 0, *c* is unbiased, with more negative values skewed toward an increasingly liberal ‘yes’ response, and more positive values indicating a conservative ‘no’. Combined, these measures are used to produce a receiver operating characteristic (ROC) curve, indicating whether an animal is capable of detecting a stimulus, and at what threshold the internal processes elicit a behavioural response.

To determine fine-scale behaviour FAR and HR from observed changes in inter-individual distance, ninety-five percent confidence intervals (CI) for the slope in a generalised least squares regression (GLS) were calculated for each individual trial over the ten-minute acoustic “exposure” period and compared to those performed across the control group average. A hit (during acoustic treatments) or false alarm (during ambient and masked controls) was identified when a trial was determined to deviate from the “normative fit”, whereby either the upper bound trial CI was less than the lower bound of the weighted control treatment effect, or the lower bound trial CI was higher than the weighted control treatment effects higher bound. The total number of correct (signal present: “hit”; signal absent: “correct non-response”), and incorrect responses (signal present: “miss”; signal absent: “false alarm”) was used to calculate fine-scale behaviour discriminability and response criterion under ambient and masked treatments.

### Statistical tests

Statistical analysis was performed using freeware programme R (v 3.2.2 and v 4.4.1) [63] using Kruskal-Wallis tests and generalised linear mixed-effects models (GLMMs), with a decision threshold (alpha) of 0.05 applied to statistical tests. Generalised least squares (GLS) regression models were also performed.

Tests were conducted to assess whether data met the assumptions for normality (Shapiro-Wilk test) and homoscedasticity (Levene’s test). To determine whether there were differences between treatments in the number of *startle responses* exhibited at the onset of tonal stimuli, binomial logistic regression analysis was performed. Kruskal-Wallis tests were used to determine whether treatment influenced: 1) the total number of individuals within a group startling at the onset of tonal stimuli, and 2) the number of undisturbed, continuous startle responses to the pulsed tonal stimuli. The Dunn-Bonferroni *post hoc* method was conducted when differences between treatments were highlighted, providing a description of where and to what extent these occurred.

For analyses of *group speed, inter-individual distance*, and *alignment*, video tracked data points were averaged (mean) to 1 s outputs and the median, and median absolute deviation were calculated over 30 s, totalling 11 blocked “time” periods. GLMMs were performed in the glmmTMB package [64], with each of the three fine-scale behaviour metrics used as a response variable in their own separate model. Treatment and time were assigned as explanatory variables. Change in response over time was included in each of the models as a random effect variable of Trial ID, with a random slope and intercept per trial to account for repeated measures from the same group of fish. As response variables could not be transformed to meet assumptions of normality before performing GLMMs, appropriate error distributions were assumed within each model. Model fits were assessed using the DHARMa package [65]. Group speed and alignment metrics were analysed with a Gamma error structure and a “log” link function while inter-individual distance was analysed with a Tweedie error and “log” link function. Chi-square statistics were calculated using the car package [66]. Finally, for each fine-scale behaviour metric, the total percentage of blocked time periods observed to deviate above or below the AMB-C median (with no overlap in median absolute deviation: MAD) were assessed per treatment (accounting for the subject-level to accommodate repeated measures, i.e., Trial ID).

For the calculation of fine-scale behaviour FAR and HR based on changes in inter-individual distance, GLS regression models were estimated for each individual trial across the ten-minute acoustic exposure period using the nlme package [67]. Ninety-five percent confidence intervals were estimated for each trial from the GLS, and compared against the average slope and confidence interval obtained from the corresponding control group (i.e., AMB-C or MASK-C). GLS deals with violations of Gauss-Markov assumptions as it does not assume that the error terms of the regression model are identically distributed, or have a constant variance. Repeated measurements over time were modelled within each trial to meet assumptions of independent sampling and account for potential autocorrelation.

## Results

### Startle response

**Fig 2:**
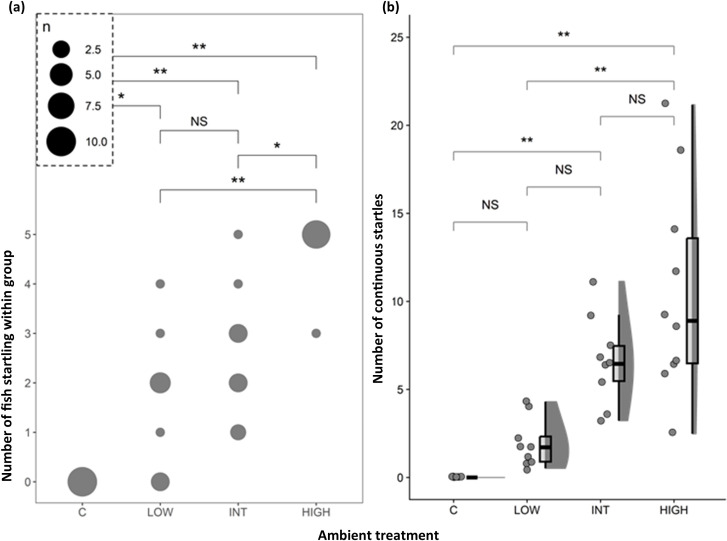
(*a*) Number of individuals within a group of five carp (*Cyprinus carpio*) exhibiting a startle response during experimental trials (n) at the onset of tonal stimuli under ambient conditions; and (*b*): number of continuous startle responses from the onset of tonal stimuli under ambient conditions, by at least one individual fish within a group of five carp: AMB-LOW (110 dB re 1 µPa), AMB-INT (121 dB re 1 µPa), and AMB-HIGH (130 dB re 1 µPa), (RMS) displayed. Note: ** indicates significance of p < 0.01; * significance of p < 0.05; and NS symbolises non-significance for Kruskal-Wallis rank sum tests ([a] χ2 = 30.88; d.f. = 3; p < 0.01 & [b] χ2 = 30.854; d.f. = 3; p < 0.01, respectively) with *post hoc* Dunn-Bonferroni tests. Note: for (b), raincloud plots show main summary statistics (i.e., boxplot with median, interquartile range, max/min), raw data (i.e., points), and kernel density estimate (i.e., shaded grey area indicating probability density function of the variable).

Fish startled at the onset of exposure to tonal stimuli under all ambient treatment SNRs (Fig 2a). The greater the intensity of the signal, the more fish within a group startled (Fig 2a: $\chi_{3}^{2}$ = 30.88; *p* < 0.01). AMB-HIGH elicited the highest number of startles (median _(IQR)_: 5 _(0)_), followed by AMB-INT (median _(IQR)_: 2.5 _(1)_), then AMB-LOW (median _(IQR)_: 2 _(1.75)_). More intense tones also stimulated a greater number of continuous startle responses under ambient treatments (Fig 2b: $\chi_{3}^{2}\;$= 31.64; *p* < 0.01). For masked treatments, no startles were observed at the onset of the acoustic stimulus with the exception of for one individual under the MASK-INT condition.

### Group swimming speed

**Fig 3:**
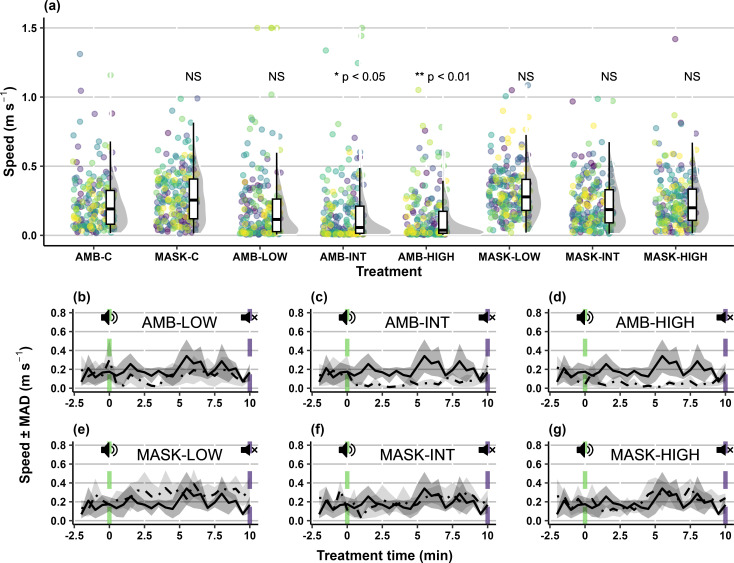
(*a*) Swimming speed (m s^-1^) for groups (n = 10 per condition) of five carp (*Cyprinus carpio*) during experimental trials under ambient and masked conditions; and median swimming speed ± median absolute deviation over time during the ambient control (“AMB-C”: solid line) and treatment (dash-dotted line) conditions: (*b*) ambient low SPL (‘AMB-LOW’); (*c*) ambient intermediate SPL (‘AMB-INT’); (*d*) ambient high SPL (‘AMB-HIGH’); (*e*) masking low SPL (‘MASK-LOW’); (*f*) masking intermediate SPL (‘MASK-INT’); and (*g*) masking high SPL (‘MASK-HIGH’). Notes: ‘SPL’ is Sound Pressure Level, while ‘MASK-C’ is the masking control. Raincloud plots show main summary statistics (i.e., boxplot with median, interquartile range, max/min), kernel density estimate (i.e., shaded grey area indicating probability density function of the variable), and raw data (i.e., points). As individual trials consist of repeated measures over time, for each condition in panel ‘a’, raw data points are shown per trial for each treatment using the viridis colour scale. In panels ‘b’ to ‘g’, vertical green dashed lines (speaker with “waves”) indicate start of the tonal exposure period and vertical purple dashed lines (speaker with an X), the end.

For masked treatments, no differences in group swimming speed were observed between control and treatment groups (Fig 3). For all ambient treatments, following a brief increase in speed (attributed to initial startle responses), carp median group swimming speed decreased by over half compared to the control trials ($\chi_{7}^{2}\;$= 41.63; *p* < 0.001; Fig 3a). The more intense the tonal stimuli, the longer and slower the swimming speed was observed to deviate from the baseline ($\chi_{1}^{2}\;$= 4.66; *p* < 0.05; Fig 3b-d). For AMB-LOW, an initial decrease in speed was observed during the exposure (median _(IQR)_: 0.11 _(0.17)_; *Z* = −1.24; *p* = 0.22; Fig 3b), but was not considered to deviate significantly from the baseline (AMB-C median _(IQR)_: 0.21 _(0.13)_). For AMB-INT group swimming speed was less than control fish for 14.3% of the tonal exposure (median _(IQR)_: 0.06 _(0.09)_; *Z* = −2.41; *p* < 0.05; Fig 3c). Group swimming speed decreased for AMB-HIGH and remained continually less for 19.3% of the exposure (median _(IQR)_: 0.03 _(0.08)_; *Z* = −3.52; *p* < 0.01; Fig 3d).

## Inter-individual distance

**Fig 4:**
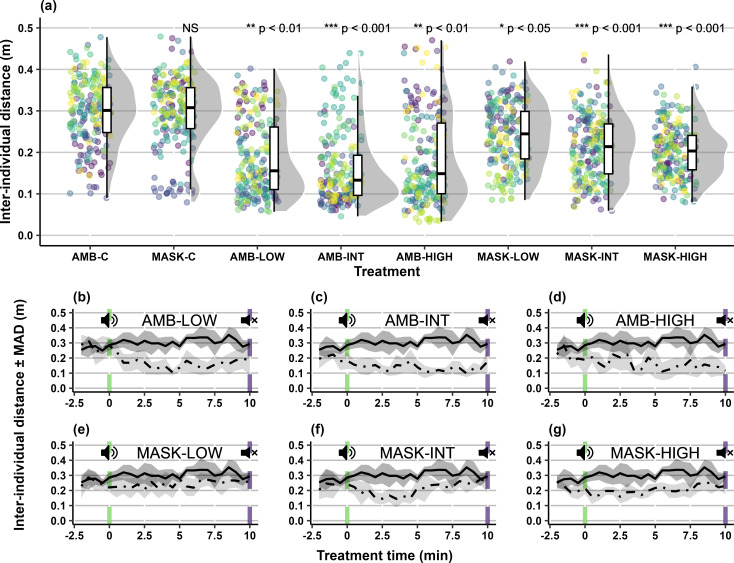
(a) Inter-individual distance (m) for groups (n = 10 per condition) of five carp (*Cyprinus carpio*) during experimental trials under ambient and masked conditions; and median inter-individual distance ± median absolute deviation over time during ambient control (AMB-C: solid line) and treatment (dash-dotted line) conditions. (*b*) ambient low SPL (‘AMB-LOW’); (*c*) ambient intermediate SPL (‘AMB-INT’); (*d*) ambient high SPL (‘AMB-HIGH’); (*e*) masking low SPL (‘MASK-LOW’); (*f*) masking intermediate SPL (‘MASK-INT’); and (*g*) masking high SPL (‘MASK-HIGH’). Notes: ‘SPL’ is Sound Pressure Level, while ‘MASK-C’ is the masking control. Raincloud plots show main summary statistics (i.e., boxplot with median, interquartile range, max/min), kernel density estimate (i.e., shaded grey area indicating probability density function of the variable), and raw data (i.e., points). As individual trials consist of repeated measures over time, for each condition in panel ‘a’, raw data points are shown per trial using the viridis colour scale. In panels ‘b’ to ‘g’, vertical green dashed lines (speaker with “waves”) indicate start of the tonal exposure period and vertical purple dashed lines (speaker with an X), the end.

Under masked treatments, differences in cohesion compared to the controls were observed, with groups exhibiting higher cohesion (i.e., a decrease in inter-individual distance) for a greater proportion of time under greater acoustic intensities (Fig 4). Group cohesion was higher under MASK-LOW during exposure (median _(IQR)_: 0.26 _(0.09)_; *Z* = −2.01; *p* < 0.05; Fig 4e), but did not deviate for any substantial period of time from the baseline (AMB-C median _(IQR)_: 0.31 _(0.04)_). Higher group cohesion was also observed for MASK-INT (median _(IQR)_: 0.22 _(0.08)_; *Z* = −3.72; *p* < 0.001; Fig 4f) in comparison to the baseline, lasting for 19% of the exposure. Higher cohesion was again observed for MASK-HIGH (median _(IQR)_: 0.20 _(0.04)_; *Z* = −3.62; *p* < 0.001; Fig 4g), with group cohesion higher for 23.8% of the exposure, in comparison to baseline behaviour. For ambient treatments, group cohesion was higher when carp were exposed to the tonal stimuli. For AMB-LOW, the inter-individual distance reduced in comparison to the baseline (median _(IQR)_: 0.17 _(0.08)_; *Z* = −2.95; *p* < 0.01; Fig 4b). For AMB-INT, higher cohesion was observed (median _(IQR)_: 0.12 _(0.07)_; *Z* = −3.99; *p* < 0.001; Fig 4c) and remained so for 66.7% of the exposure, in comparison to the baseline. Groups exposed to AMB-HIGH also displayed higher cohesion (median _(IQR)_: 0.14 _(0.14)_; *Z* = −2.61; *p* < 0.01; Fig 4d), lasting for 38.1% of the comparative exposure.

### Alignment

**Fig 5:**
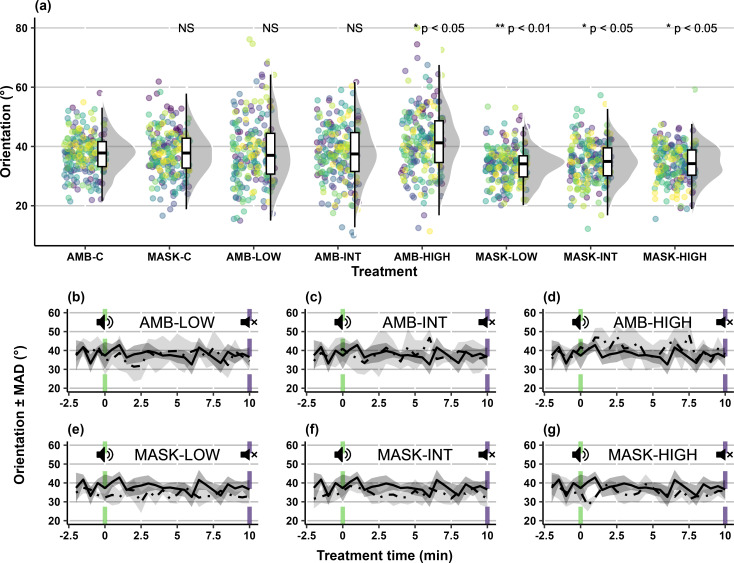
(*a*) Orientation (°) for groups (n = 10 per condition) of five carp (*Cyprinus carpio*) during experimental trials under ambient and masked conditions; and median orientation ± median absolute deviation over time during ambient control (AMB-C: solid line) and treatment (dash-dotted line) conditions: (*b*) ambient low SPL (‘AMB-LOW’); (*c*) ambient intermediate SPL (‘AMB-INT’); (*d*) ambient high SPL (‘AMB-HIGH’); (*e*) masking low SPL (‘MASK-LOW’); (*f*) masking intermediate SPL (‘MASK-INT’); and (*g*) masking high SPL (‘MASK-HIGH’). Notes: ‘SPL’ is Sound Pressure Level, while ‘MASK-C’ is the masking control. Lower orientation values represent greater alignment. Raincloud plots show main summary statistics (i.e., boxplot with median, interquartile range, max/min), kernel density estimate (i.e., shaded grey area indicating probability density function of the variable), and raw data (i.e., points). As individual trials consist of repeated measures over time, for each condition in panel ‘a’, raw data points are shown per trial using the viridis colour scale. In panels ‘b’ to ‘g’, vertical green dashed lines (speaker with “waves”) indicate start of the tonal exposure period and vertical purple dashed lines (speaker with an X), the end.

Differences in orientation were observed between groups exposed to masked treatments ${(\chi }_{7}^{2}\;$= 40.74; *p* < 0.001), with fish becoming increasingly aligned in the same direction in the presence of the tonal stimuli. However, there was no linear relationship between alignment and acoustic intensity (Fig 5), or effect of time ${(\chi }_{1}^{2}\;$= 1.81; *p* = 0.18). Groups experiencing MASK-LOW became more aligned during exposure (median _(IQR)_: 33.4 _(2.83)_; *Z* = −2.88; *p* < 0.01; Fig 5e) and did so for 4.76% of the duration. For MASK-INT, alignment increased from the baseline (median _(IQR)_: 34.8 _(1.94)_; *Z* = −2.06; *p* < 0.05; Fig 5f), but did not deviate for any substantial period of time from the baseline (AMB-C median _(IQR)_: 37.8 _(2.42)_). Finally, MASK-HIGH groups exhibited increased alignment (median _(IQR)_: 33.8 _(2.45)_; *Z* = −2.53; *p* < 0.05; Fig 5g) and did so for 9.52% of the exposure period. Under all ambient treatments, median group orientation initially decreased in response to tonal stimuli as individuals became more aligned with one another, but quickly returned to the baseline. For AMB-HIGH, this secondary shift in orientation involved fish decreasing their alignment in comparison to control groups. Group alignment differed from the baseline for AMB-HIGH (median _(IQR)_: 41.4 _(4.73)_; *Z* = 2.38; *p* < 0.05; Fig 5g), but not for AMB-INT (median _(IQR)_: 38.1 _(3.93)_; *Z* = −0.11; *p* = 0.91; Fig 5f) or AMB-LOW (median _(IQR)_: 36.9 _(4.38)_; *Z* = 0.39; *p* = 0.69; Fig 5e), but the deviation was not for any substantial period of time from the baseline.

### Signal detection

**Fig 6:**
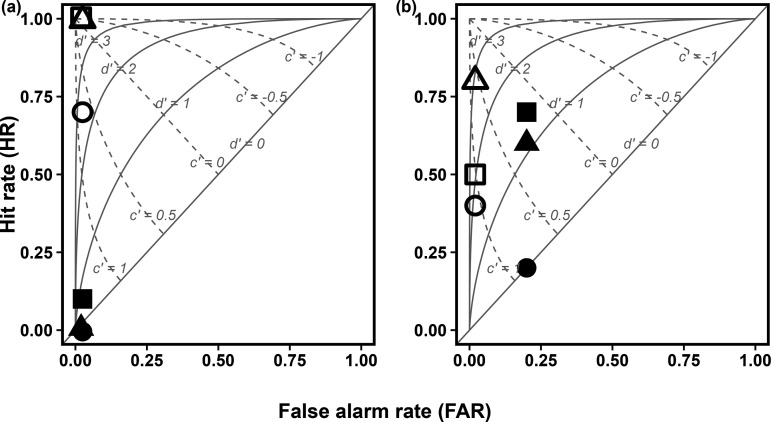
Receiver-operating characteristics (ROC) plots of hit rate against false-alarm rate for masked. (●,■,▲) and ambient (○,□,△) treatment (*a*) coarse-scale (startle response); and (*b*) fine-scale (inter-individual distance) behavioural responses of *Cyprinus carpio* to onset of a tonal acoustic stimuli at low SPL (‘LOW’●,○); intermediate SPL (‘INT’■,□); and high SPL (‘HIGH’▲,△) signal-to-noise ratio (SNR). Solid light grey lines indicate reference discriminability (*d’* = 0, 1, 2, 3), with an increase in *d’* representing a greater signal discriminability. Dashed lines show response criterion (*c* = −1, −0.5, 0, 0.5, 1), with an increase in *c* representing a greater bias toward responding. Note: ‘SPL’ is Sound Pressure Level.

Coarse-scale (startle response) signal discriminability was lower under masked (MASK-LOW: *d’* = 0; MASK-INT: *d’* = 0.36; MASK-HIGH: *d’* = 0; Fig 6a) than ambient treatments (AMB-LOW: *d’* = 2.17; AMB-INT: *d’* = 3.29; AMB-HIGH: *d’* = 3.29; Fig 6a), with an increase in discriminability observed at higher signal strengths. While the coarse-scale response criterion was positive for all treatments, groups exposed to masked treatments tended not to startle (MASK-LOW: *c* = 1.64; MASK-INT: *c* = 1.46; MASK-HIGH: *c* = 1.64; Fig 6a), whereas those under ambient treatments exhibited a reasonably unbiased response criterion (AMB-LOW: *c* = 0.56; AMB-INT: *c* = 5.55e^-16^; AMB-HIGH: *c* = 5.55e^-16^; Fig 6a).

Fine-scale signal discriminability was lower under masked (MASK-LOW: *d’* = 0; MASK-INT: *d’* = 1.37; MASK-HIGH: *d’* = 1.10; Fig 6b; S1 Table) compared with ambient treatments (AMB-LOW: *d’* = 1.39; AMB-INT: *d’* = 1.64; AMB-HIGH: *d’* = 2.49; Fig 6b; S2 Table). Discriminability was observed to increase at greater signal strengths (relative to noise) under both conditions. Response criterion for fine-scale behaviour were positive and reasonably unbiased under masked treatments (MASK-LOW: *c* = 0.84; MASK-INT: *c* = 0.16; MASK-HIGH: *c* = 0.29; Fig 6b), with a lower response likelihood for the MASK-LOW treatment. For ambient treatments, response criterion were unbiased, but were more conservative (positive) than for coarse-scale responses (AMB-LOW: *c* = 0.95; AMB-INT: *c* = 0.82; AMB-HIGH: *c* = 0.40; Fig 6b).

## Discussion

This study provides insights on how masking noise influences fish response to acoustic signals and how the interpretation of results can depend on the methodological approach adopted and scale of behaviour exhibited. We tested three predictions related to the influence of acoustic masking on the response of groups of five juvenile common carp to tonal signals that differed in intensity. First, we examined coarse (startle) and fine-scale (swimming speed, inter-individual distance, and alignment) behaviours and predicted that these would diminish when the signal was masked, compared to an ambient control. Under ambient background noise, carp clearly startled when exposed to a tonal signal, swam slower, and became more aligned in more cohesive groups. As predicted, these responses were lower when a broadband noise was used to mask the signal, with startles largely absent, and fine-scale responses suppressed although positively related to the signal-to-noise ratio (SNR). In support of our second prediction, both swimming speed and alignment deviated from the baseline behaviour for longer under ambient than masked treatments, while higher SNR treatments elicited a greater number of continuous startles over time by at least one individual fish within a group. Finally, through the application of SDT we predicted that the probability of signal detection would be lower, and the likelihood of an incorrect ‘false alarm’ or ‘miss’ higher, when the signal was masked. Under ambient noise conditions, the false alarm rate for groups of carp exposed to a tonal stimulus was zero, and signal discriminability was high and positively correlated with an increasing signal to noise ratio. In support of our prediction, the false alarm rate was higher under masking noise, and signal discriminability was lower.

Under ambient background noise, all acoustic treatments elicited a startle response in one or more subject fish. A startle response, in which a fish contracts its body before burst swimming along an erratic path, is commonly observed in response to a perceived threat [68–72]. In this study we built on previous work (*e.g.,* [26]) by accommodating collective behaviour in the analysis of the response of a species that commonly aggregates. Both broadband (e.g., seismic air gun [73]; pulsed white noise [61]) and simple sinewave tones (e.g*.,* 0.1–2 kHz [69]; 150 Hz and 2.2 kHz [25]) are known to induce a group level startle response in both marine (e.g., European seabass, *Dicentrarchus labrax*, thicklip mullet, *Chelon labrosus* [69]) and freshwater fish species (e.g., zebrafish, *Danio rerio* [61]; Eurasian minnow [24–26]). In this study, the number of group members that startled was positively related to acoustic intensity, supporting the observations of others, both under laboratory (e.g*.*, goldfish, *Carassius auratus* [68]) and field settings (e.g*.,* European seabass [70]).

In contrast to the ambient background noise conditions presented, startles were absent under all the masked treatments, except for a single event. To some extent a lack of a response was unexpected, based on the available information on the hearing ability of common carp [51,46]. Relying on startle responses alone would prevent us from determining whether this lack of a response reflects the inability of the subject fish to discriminate the signal from the noise, based on the specific sensitivity of the auditory system, or an ability to do so followed by a failure to make sense of the information gained and to respond in a way deemed appropriate by the observer. This highlights the two stages of a process operating at the levels of both the subject fish and experimentalist. As such, conclusions formed by considering coarse-scale responses alone may be misleading, especially when the value of exploring fine-scale ecologically relevant responses of fish to sound have previously been described (e.g., alteration of: coordinated movement [50]; spatial distribution [25]; and orientation and cohesion of groups [24]). Consideration of more nuanced behaviours allow for a more in-depth analysis of group-level responses to be undertaken to enhance understanding of the influence of sound fields and acoustic masking.

When considering fine-scale responses, it appears the fish were able to detect the signal despite masking, at least under the higher SPLs, as indicated by groups becoming more cohesive compared to the controls. However, fine-scale response to the tonal signal varied, with limited differences between the masked treatment and control when considering swimming speed and group alignment. In the absence of masking, groups swam slower over time and became more tightly packed (particularly at higher SPLs) when exposed to the stimulus, possibly indicating a fear or anxiety-like behaviour associated with an increasing perception of threat [74]. An association between swimming speed and adjustments in inter-individual distance has previously been observed [75], and reductions in movement [76] and increased cohesion may reduce predation risk [77,78] and enhance information sharing [50].

Using SDT, signal discriminability as indicated by fine-scale responses was lower under the masked treatments than for ambient noise. However, signals become more discriminable with increasing SNR, and a positive response bias became less conservative. The use of SDT is valuable in evaluating fine-scale responsiveness of animals to stimuli, supporting the information obtained from more coarse-scale analysis, because it accounts for differences in internal status that may be influenced by innumerable factors, such as those related to physiological status, prior experience and behavioural phenotype [38,79].

In this study, a small tank set-up allowed a carefully controlled reductionist approach to be adopted to minimise the influence of confounding factors, and provide a stable, reproducible acoustic field. As expected, owing to the nature of the near-field conditions relative to wavelength, particle motion was complex and highly variable in all directions [80]. This was not considered an issue as the main parameters of interest was the behavioural responses of carp (a pressure-sensitive otophysine fish) to tonal stimuli under masked and ambient noise conditions at a known SNR in the sound pressure domain. The relationship between the pressure and particle motion components of sound is understood to differ between those generated within small tank setups and large-scale “natural” aquatic habitats (e.g*.*, deep lakes or oceans [24]). Owing to the small dimensions of the tank, the material properties of the walls (influencing resonance frequencies), and the sound speed differences between water and the surrounding air, high levels of particle motion are produced within the sound field [81]. In contrast, the acoustic nature of shallow streams (commonly < 1 m depth), man-made flowing channels, or rivers, are more convoluted, and not well understood [24,82,83]. Extrapolation of results to natural systems should therefore undergo appropriate field study validation.

Understanding how fish detect and respond to sound and what factors influence this is important in application of fundamental knowledge to fisheries management and conservation. On one hand, such information may be used in the design of more efficient acoustic deterrents [24,25,71,72], such as those used to guide fish away from dangerous areas that may compromise fitness (e.g., water abstraction points or turbine intakes [84]), or in the case of invasive species, providing attraction to traps so that they may be removed from the system [85]. On the other hand, greater understanding will help predict the potential impacts of globally rising levels of anthropogenic noise [20]. Previous studies of fish response to sound tended to neglect the influence of background noise on behaviours exhibited, focusing instead primarily on the characteristics of the signal (see [45,46,48] for some exceptions). Likewise, reductive experimental approaches often consider only individual fish, failing to recognise that many migratory species aggregate, shoal or school (see [26] for a comparative assessment of group and individual responses to human-generated sound). Further efforts are required to address these biases. Finally, expanding this research to consider interactions between multimodal stimuli and how a masking factor in one modality influences signal detection in the other (e.g., [39]) is likely to provide important insight of value in advancing environment impact mitigation technology.

## Supporting information

S1 Fig Insonified experimental area showing Sound Pressure Levels (RMS) (dB re 1 µPa) recorded at (*a*) 7 cm, (*b*) 13.5 cm, and (*c*) 20 cm water depth for (*i*) 170 Hz (sinewave) tonal treatment; and 120–3000 Hz broadband masking noise recorded (*ii*) across the broadband noise frequency range, and (*iii*) within the 1/3^rd^ octave band. Note: points indicate hydrophone matrix positioning.
(TIF)

S2 Fig Heat maps of particle acceleration (dB re 1 mm s^-2^) measured at 13.5 cm depth for (a) 170 Hz sinewave tone; and (b) broadband noise (120–3000 Hz). (TIF)

S1 Table False alarm (FAR) and hit rates (HR) for trial groups exposed to 170 Hz tonal stimuli under masked noise treatments determined through the calculation of generalised least squares regression models. Note: Grey shading indicates that a trial (01−10) deviated from the group “normative fit” (regression line equation: y = 0.304–4.8 x 10^−6^ x; ± s.e. = ± 2.14 x 10^−5^; CI [−4.68 x 10^−5^; 3.72 x 10^−5^]) and was classed as a “false alarm” (incorrect response for control) or “hit” (correct response for treatments).
(DOCX)

S2 Table False alarm (FAR) and hit rates (HR) for trial groups exposed to 170 Hz tonal stimuli under ambient noise treatments determined through the calculation of generalised least squares regression models. *Note*: Grey shading indicates that a trial (01−10) deviated from the group “normative fit” (regression line equation: y = 0.217 + 3.84 x 10^−5^ x; ± s.e. = ± 2.04 x 10^−5^; CI [−1.70 x 10^−6^; 7.84 x 10^−5^]) and was classed as a “false alarm” (incorrect response for control) or “hit” (correct response for treatments).
(DOCX)

## References

[1] Catchpole CK, Slater PJB. Bird song: biological themes and variations. 2nd ed. New York, NY, US: Cambridge University Press. 2008. 335, xi.

[2] Feng AS, Narins PM, Xu C-H, Lin W-Y, Yu Z-L, Qiu Q, et al. Ultrasonic communication in frogs. Nature. 2006;440(7082):333–6. doi: 10.1038/nature04416 16541072

[3] Nakano R, Takanashi T, Surlykke A. Moth hearing and sound communication. J Comp Physiol A. 2015;201(1):111–21. doi: 10.1007/s00359-014-0945-8 25261361

[4] Amoser S, Ladich F. Year-round variability of ambient noise in temperate freshwater habitats and its implications for fishes. Aquat Sci. 2010;72(3):371–8. doi: 10.1007/s00027-010-0136-9 20922061 PMC2948566

[5] Swanson FJ, Kratz TK, Caine N, Woodmansee RG. Landform effects on ecosystem patterns and processes. BioScience. 1988;38(2):92–8. doi: 10.2307/1310614

[6] Pijanowski BC, Villanueva-Rivera LJ, Dumyahn SL, Farina A, Krause BL, Napoletano BM, et al. Soundscape ecology: the science of sound in the landscape. BioScience. 2011;61(3):203–16. doi: 10.1525/bio.2011.61.3.6

[7] Sueur J, Farina A. Ecoacoustics: the ecological investigation and interpretation of environmental sound. Biosemiotics. 2015;8(3):493–502. doi: 10.1007/s12304-015-9248-x

[8] Farina A. Principles and methods in landscape ecology: Towards a science of the landscape. 2nd ed. Dordrecht: Springer. 2006.

[9] Bayne EM, Habib L, Boutin S. Impacts of chronic anthropogenic noise from energy-sector activity on abundance of songbirds in the boreal forest. Conserv Biol. 2008;22(5):1186–93. doi: 10.1111/j.1523-1739.2008.00973.x 18616740

[10] Ellison WT, Southall BL, Clark CW, Frankel AS. A new context-based approach to assess marine mammal behavioral responses to anthropogenic sounds. Conserv Biol. 2012;26(1):21–8. doi: 10.1111/j.1523-1739.2011.01803.x 22182143

[11] Bruintjes R, Radford AN. Context-dependent impacts of anthropogenic noise on individual and social behaviour in a cooperatively breeding fish. Anim Behav. 2013;85(6):1343–9. doi: 10.1016/j.anbehav.2013.03.025

[12] Simpson SD, Meekan M, Montgomery J, McCauley R, Jeffs A. Homeward sound. Science. 2005;308(5719):221. doi: 10.1126/science.1107406 15821083

[13] Bass AH, McKibben JR. Neural mechanisms and behaviors for acoustic communication in teleost fish. Prog Neurobiol. 2003;69(1):1–26. doi: 10.1016/s0301-0082(03)00004-2 12637170

[14] Amorim MCP, Stratoudakis Y, Hawkins AD. Sound production during competitive feeding in the grey gurnard. J Fish Biol. 2004;65(1):182–94. doi: 10.1111/j.0022-1112.2004.00443.x

[15] Moulton JM. Swimming sounds and the schooling of fishes. Biol Bull. 1960;119(2):210–23. doi: 10.2307/1538923

[16] Ward D, Morison F, Morrissey E, Jenks K, Watson WH 3rd. Evidence that potential fish predators elicit the production of carapace vibrations by the American lobster. J Exp Biol. 2011;214(Pt 15):2641–8. doi: 10.1242/jeb.057976 21753058

[17] Cox K, Brennan LP, Gerwing TG, Dudas SE, Juanes F. Sound the alarm: a meta-analysis on the effect of aquatic noise on fish behavior and physiology. Glob Chang Biol. 2018;24(7):3105–16. doi: 10.1111/gcb.14106 29476641

[18] Davies HL, Cox KD, Murchy KA, Shafer HM, Looby A, Juanes F. Marine and freshwater sounds impact invertebrate behavior and physiology: a meta-analysis. Glob Chang Biol. 2024;30(11):e17593. doi: 10.1111/gcb.17593 39582363 PMC11586707

[19] Duarte CM, Chapuis L, Collin SP, Costa DP, Devassy RP, Eguiluz VM, et al. The soundscape of the Anthropocene ocean. Science. 2021;371(6529):eaba4658. doi: 10.1126/science.aba4658 33542110

[20] Slabbekoorn H, Bouton N, van Opzeeland I, Coers A, ten Cate C, Popper AN. A noisy spring: the impact of globally rising underwater sound levels on fish. Trends Ecol Evol. 2010;25(7):419–27. doi: 10.1016/j.tree.2010.04.005 20483503

[21] Putland RL, Merchant ND, Farcas A, Radford CA. Vessel noise cuts down communication space for vocalizing fish and marine mammals. Glob Chang Biol. 2018;24(4):1708–21. doi: 10.1111/gcb.13996 29194854

[22] Solan M, Hauton C, Godbold JA, Wood CL, Leighton TG, White P. Anthropogenic sources of underwater sound can modify how sediment-dwelling invertebrates mediate ecosystem properties. Sci Rep. 2016;6:20540. doi: 10.1038/srep20540 26847483 PMC4742813

[23] Crovo JA, Mendonça MT, Holt DE, Johnston CE. Stress and auditory responses of the otophysan fish, cyprinella venusta, to road traffic noise. PLoS One. 2015;10(9):e0137290. doi: 10.1371/journal.pone.0137290 26398211 PMC4580447

[24] Currie HAL, White PR, Leighton TG, Kemp PS. Group behavior and tolerance of Eurasian minnow (Phoxinus phoxinus) in response to tones of differing pulse repetition rate. J Acoust Soc Am. 2020;147(3):1709. doi: 10.1121/10.0000910 32237844

[25] Currie HAL, White PR, Leighton TG, Kemp PS. Collective behaviour of the European minnow (Phoxinus phoxinus) is influenced by signals of differing acoustic complexity. Behav Processes. 2021;189:104416. doi: 10.1016/j.beproc.2021.104416 33971249

[26] Short M, White PR, Leighton TG, Kemp PS. Influence of acoustics on the collective behaviour of a shoaling freshwater fish. Freshw Biol. 2020;65(12):2186–95. doi: 10.1111/fwb.13612

[27] de Jong K, Amorim MCP, Fonseca PJ, Fox CJ, Heubel KU. Noise can affect acoustic communication and subsequent spawning success in fish. Environ Pollut. 2018;237:814–23. doi: 10.1016/j.envpol.2017.11.003 29146199

[28] Sebastianutto L, Picciulin M, Costantini M, Ferrero EA. How boat noise affects an ecologically crucial behaviour: the case of territoriality in Gobius cruentatus (Gobiidae). Environ Biol Fish. 2011;92(2):207–15. doi: 10.1007/s10641-011-9834-y

[29] Holles S, Simpson S, Radford A, Berten L, Lecchini D. Boat noise disrupts orientation behaviour in a coral reef fish. Mar Ecol Prog Ser. 2013;485:295–300. doi: 10.3354/meps10346

[30] Simpson SD, Purser J, Radford AN. Anthropogenic noise compromises antipredator behaviour in European eels. Glob Chang Biol. 2015;21(2):586–93. doi: 10.1111/gcb.12685 25098970

[31] Hasan MR, Crane AL, Ferrari MCO, Chivers DP. A cross-modal effect of noise: the disappearance of the alarm reaction of a freshwater fish. Anim Cogn. 2018;21(3):419–24. doi: 10.1007/s10071-018-1179-x 29637467

[32] Schaf B. Chapter Five - Critical Bands. In: Tobias JV, editor. Foundations of Modern Auditory Theory. Academic Press; 1970. p. 157–202. doi: 10.1016/B978-0-12-691901-1.50010-3

[33] Rogers PH, Hawkins AD, Popper AN, Fay RR, Gray MD. Parvulescu revisited: small tank acoustics for bioacousticians. Adv Exp Med Biol. 2016;875:933–41. doi: 10.1007/978-1-4939-2981-8_115 26611052

[34] Ryan MJ, Brenowitz EA. the role of body size, phylogeny, and ambient noise in the evolution of bird song. Am Nat. 1985;126(1):87–100. doi: 10.1086/284398

[35] Rosa P, Koper N. Integrating multiple disciplines to understand effects of anthropogenic noise on animal communication. Ecosphere. 2018;9(2):e02127. doi: 10.1002/ecs2.2127

[36] Stanislaw H, Todorov N. Calculation of signal detection theory measures. Behav Res Methods Instrum Comput. 1999;31(1):137–49. doi: 10.3758/bf03207704 10495845

[37] Wickens TD. Elementary signal detection theory. Oxford University Press; 2001.

[38] Kemp PS, Anderson JJ, Vowles AS. Quantifying behaviour of migratory fish: application of signal detection theory to fisheries engineering. Ecol Eng. 2012;41:22–31. doi: 10.1016/j.ecoleng.2011.12.013

[39] Kerr JR, Kemp PS. Masking a fish’s detection of environmental stimuli: application to improving downstream migration at river infrastructure. J Fish Biol. 2019;95(1):228–37. doi: 10.1111/jfb.13812 30251260 PMC7379693

[40] Johnson N, Kang J, Hathway EA. Acoustics of weirs: Potential implications for micro-hydropower noise. Renew Energy. 2014;71:351–60. doi: 10.1016/j.renene.2014.05.049

[41] Miyamoto RT, McConnell SO, Anderson JJ, Feist BE. Underwater noise generated by Columbia River hydroelectric dams. J Acoust Soc Am. 1989;85(S1):S127–S127. doi: 10.1121/1.2026716

[42] Wiley RH. Errors, exaggeration, and deception in animal communication. In: Real LA, editor. Behavioural mechanisms in evolutionary ecology. University of Chicago Press. 1994.

[43] Fay RR, Ahroon WA, Orawski AA. Auditory masking patterns in the goldfish (Carassius auratus): psychophysical tuning curves. J Exp Biol. 1978;74:83–100. doi: 10.1242/jeb.74.1.83 670874

[44] Fay RR, Coombs S. Neural mechanisms in sound detection and temporal summation. Hear Res. 1983;10(1):69–92. doi: 10.1016/0378-5955(83)90018-7 6841279

[45] Buerkle U. Relation of pure tone thresholds to background noise level in the atlantic cod (Gadus morhua). J Fish Res Bd Can. 1968;25(6):1155–60. doi: 10.1139/f68-101

[46] Amoser S, Ladich F. Are hearing sensitivities of freshwater fish adapted to the ambient noise in their habitats?. J Exp Biol. 2005;208(Pt 18):3533–42. doi: 10.1242/jeb.01809 16155225

[47] Fay RR. Signal-to-noise ratio for source determination and for a comodulated masker in goldfish, Carassius auratus. J Acoust Soc Am. 2011;129(5):3367–72. doi: 10.1121/1.3562179 21568437 PMC3108397

[48] Tavolga WN. Signal-noise ratio and the critical band in fishes. J Acoust Soc Am. 1974;55(6):1323–33. doi: 10.1121/1.1914704 4846728

[49] Jacobsen L, Baktoft H, Jepsen N, Aarestrup K, Berg S, Skov C. Effect of boat noise and angling on lake fish behaviour. J Fish Biol. 2014;84(6):1768–80. doi: 10.1111/jfb.12395 24813930

[50] Herbert-Read JE, Kremer L, Bruintjes R, Radford AN, Ioannou CC. Anthropogenic noise pollution from pile-driving disrupts the structure and dynamics of fish shoals. Proc Biol Sci. 2017;284(1863):20171627. doi: 10.1098/rspb.2017.1627 28954915 PMC5627215

[51] Kojima T, Ito H, Komada T, Taniuchi T, Akamatsu T. Measurements of auditory sensitivity in common carp Cyprinus carpio by the auditory brainstem response technique and cardiac conditioning method. Fisheries Sci. 2005;71(1):95–100. doi: 10.1111/j.1444-2906.2005.00935.x

[52] Freyhof J, Kottelat M. The IUCN Red List of Threatened Species 2008: e.T6181A12559362. 2008. Cyprinus carpio.

[53] Vilizzi L, Thwaites LA, Smith BB, Nicol JM, Madden CP. Ecological effects of common carp (Cyprinus carpio) in a semi-arid floodplain wetland. Mar Freshwater Res. 2014;65(9):802. doi: 10.1071/mf13163

[54] Crichigno S, Cordero P, Blasetti G, Cussac V. Dispersion of the invasive common carp Cyprinus carpio in southern South America: changes and expectations, westward and southward. J Fish Biol. 2016 Jul 1;89(1):403–16. doi: 10.1111/jfb.12969 27095064

[55] Stuart IG, Conallin AJ. Control of globally invasive common carp: an 11-year commercial trial of the Williams’ Cage. N Am J Fish Manag. 2018;38(5):1160–9. doi: 10.1002/nafm.10221

[56] Huntingford FA, Andrew G, Mackenzie S, Morera D, Coyle SM, Pilarczyk M, et al. Coping strategies in a strongly schooling fish, the common carp Cyprinus carpio. J Fish Biol. 2010;76(7):1576–91. doi: 10.1111/j.1095-8649.2010.02582.x 20557617

[57] Fay RR. Hearing in vertebrates: a psychophysics databook. Hill-Fay Associates. 1988.

[58] Ainslie MA, Halvorsen MB, Robinson SP. A terminology standard for underwater acoustics and the benefits of international standardization. IEEE J Oceanic Eng. 2022;47(1):179–200. doi: 10.1109/joe.2021.3085947

[59] Nedelec SL, Ainslie MA, Andersson M, Sei-Him C, Halvorsen MB, Linné M, et al. Best practice guide for underwater particle motion measurement for biological applications. Technical report by the University of Exeter for the IOGP Marine Sound and Life Joint Industry Programme. 2021.

[60] Bhandiwad AA, Zeddies DG, Raible DW, Rubel EW, Sisneros JA. Auditory sensitivity of larval zebrafish (Danio rerio) measured using a behavioral prepulse inhibition assay. J Exp Biol. 2013;216(Pt 18):3504–13. doi: 10.1242/jeb.087635 23966590 PMC3749908

[61] Neo YY, Parie L, Bakker F, Snelderwaard P, Tudorache C, Schaaf M, et al. Behavioral changes in response to sound exposure and no spatial avoidance of noisy conditions in captive zebrafish. Front Behav Neurosci. 2015;9:28. doi: 10.3389/fnbeh.2015.00028 25741256 PMC4330796

[62] Swets JA. Signal Detection Theory and ROC Analysis in Psychology and Diagnostics. Psychology Press. 1996.

[63] R Core Team. R: A language and environment for statistical computing. Vienna, Austria: R Foundation for Statistical Computing. 2024.

[64] McGillycuddy M, Warton DI, Popovic G, Bolker BM. Parsimoniously fitting large multivariate random effects in glmmTMB. J Stat Soft. 2025;112(1). doi: 10.18637/jss.v112.i01

[65] Hartig F. DHARMa: residual diagnostics for hierarchical (Multi-Level/Mixed) regression models. The Comprehensive R Archive Network (CRAN), R package version 0.4.7. https://cran.r-project.org/web/packages/DHARMa/index.html (accessed Apr. 2025).

[66] Fox J, Weisberg S. An R companion to applied regression: Appendices. Second. Robust regression in R. Thousand Oaks (CA): Sage; 2014.

[67] Pinheiro J, Bates D, R Core Team. Nlme: linear and nonlinear mixed effects models. R package version 3.1-168. https://CRAN.R-project.org/package=nlme (accessed May. 2025).

[68] Domenici P, Blake R. The kinematics and performance of fish fast-start swimming. J Exp Biol. 1997;200(Pt 8):1165–78. doi: 10.1242/jeb.200.8.1165 9319004

[69] Kastelein RA, Heul S van der, Verboom WC, Jennings N, Veen J van der, de Haan D. Startle response of captive North Sea fish species to underwater tones between 0.1 and 64 kHz. Mar Environ Res. 2008;65(5):369–77. doi: 10.1016/j.marenvres.2008.01.001 18295877

[70] Kastelein RA, Jennings N, Kommeren A, Helder-Hoek L, Schop J. Acoustic dose-behavioral response relationship in sea bass (Dicentrarchus labrax) exposed to playbacks of pile driving sounds. Mar Environ Res. 2017;130:315–24. doi: 10.1016/j.marenvres.2017.08.010 28874258

[71] Holgate A, White PR, Leighton TG, Kemp P. Adopting a reductionist approach to advance acoustic deterrents in fish conservation. Front Freshw Sci. 2024;2. doi: 10.3389/ffwsc.2024.1320582

[72] Holgate A, White PR, Leighton TG, Kemp PS. Applying appropriate frequency criteria to advance acoustic behavioural guidance systems for fish. Sci Rep. 2023;13(1):8075. doi: 10.1038/s41598-023-33423-5 37202429 PMC10195784

[73] Wardle CS, Carter TJ, Urquhart GG, Johnstone ADF, Ziolkowski AM, Hampson G, et al. Effects of seismic air guns on marine fish. Cont Shelf Res. 2001;21(8–10):1005–27. doi: 10.1016/s0278-4343(00)00122-9

[74] Neo YY, Seitz J, Kastelein RA, Winter HV, ten Cate C, Slabbekoorn H. Temporal structure of sound affects behavioural recovery from noise impact in European seabass. Biol Conserv. 2014;178:65–73. doi: 10.1016/j.biocon.2014.07.012

[75] Kent MIA, Lukeman R, Lizier JT, Ward AJW. Speed-mediated properties of schooling. R Soc Open Sci. 2019;6(2):181482. doi: 10.1098/rsos.181482 30891275 PMC6408369

[76] Anholt BR, Werner E, Skelly DK. effect of food and predators on the activity of four larval ranid frogs. Ecology. 2000;81(12):3509–21. doi: 10.1890/0012-9658(2000)081[3509:eofapo]2.0.co;2

[77] Handegard NO, Boswell KM, Ioannou CC, Leblanc SP, Tjøstheim DB, Couzin ID. The dynamics of coordinated group hunting and collective information transfer among schooling prey. Curr Biol. 2012;22(13):1213–7. doi: 10.1016/j.cub.2012.04.050 22683262

[78] Ioannou CC, Guttal V, Couzin ID. Predatory fish select for coordinated collective motion in virtual prey. Science. 2012;337(6099):1212–5. doi: 10.1126/science.1218919 22903520

[79] Jolles JW, King AJ, Killen SS. The role of individual heterogeneity in collective animal behaviour. Trends Ecol Evol. 2020;35(3):278–91. doi: 10.1016/j.tree.2019.11.001 31879039

[80] Gray M, Rogers PH, Zeddies DG. Acoustic particle motion measurement for bioacousticians: principles and pitfalls. Proc Mtgs Acoust. 2016; 27:010022. doi: 10.1121/2.0000290

[81] Akamatsu T, Okumura T, Novarini N, Yan HY. Empirical refinements applicable to the recording of fish sounds in small tanks. J Acoust Soc Am. 2002;112(6):3073–82. doi: 10.1121/1.1515799 12509030

[82] Campbell J, Shafiei Sabet S, Slabbekoorn H. Particle motion and sound pressure in fish tanks: a behavioural exploration of acoustic sensitivity in the zebrafish. Behav Processes. 2019;164:38–47. doi: 10.1016/j.beproc.2019.04.001 30953790

[83] Leighton TG, Currie HAL, Holgate A, Dolder CN, Jones SL, White PR, et al. Analogies in contextualizing human response to airborne ultrasound and fish response to acoustic noise and deterrents. Proc Mtgs Acoust. 2020 Jun 19;37(1):010014. doi: 10.1121/2.0001260

[84] Piper AT, White PR, Wright RM, Leighton TG, Kemp PS. Response of seaward-migrating European eel (Anguilla anguilla) to an infrasound deterrent. Ecol Eng. 2019;127:480–6. doi: 10.1016/j.ecoleng.2018.12.00

[85] Isabella-Valenzi L, Higgs DM. Development of an acoustic trap for potential round goby (Neogobius melanostomus) management. J Great Lakes Res. 2016;42(4):904–9. doi: 10.1016/j.jglr.2016.05.004

