# Supplementary figures and images for: Masking noise reduces the anti-predator-like response to an acoustic stimulus: Application of Signal Detection Theory to fish behaviour
Source: PLoS One. 2025 Jul 11;20(7):e0327092. doi: 10.1371/journal.pone.0327092 (PMC12250208; doi:10.1371/journal.pone.0327092)

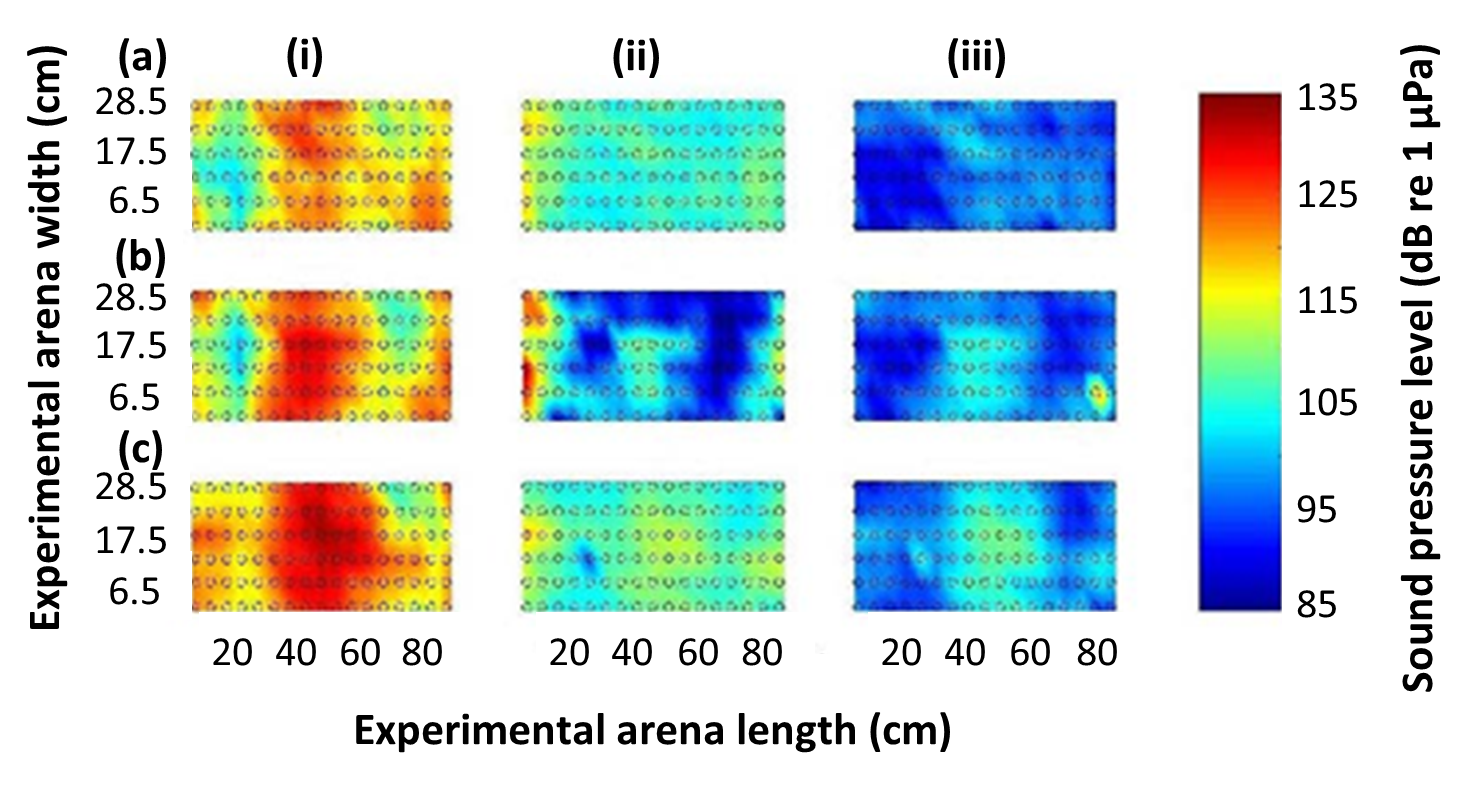

Supplement: S1 Fig — Note: points indicate hydrophone matrix positioning. (TIF) [file pone.0327092.s001.tif]

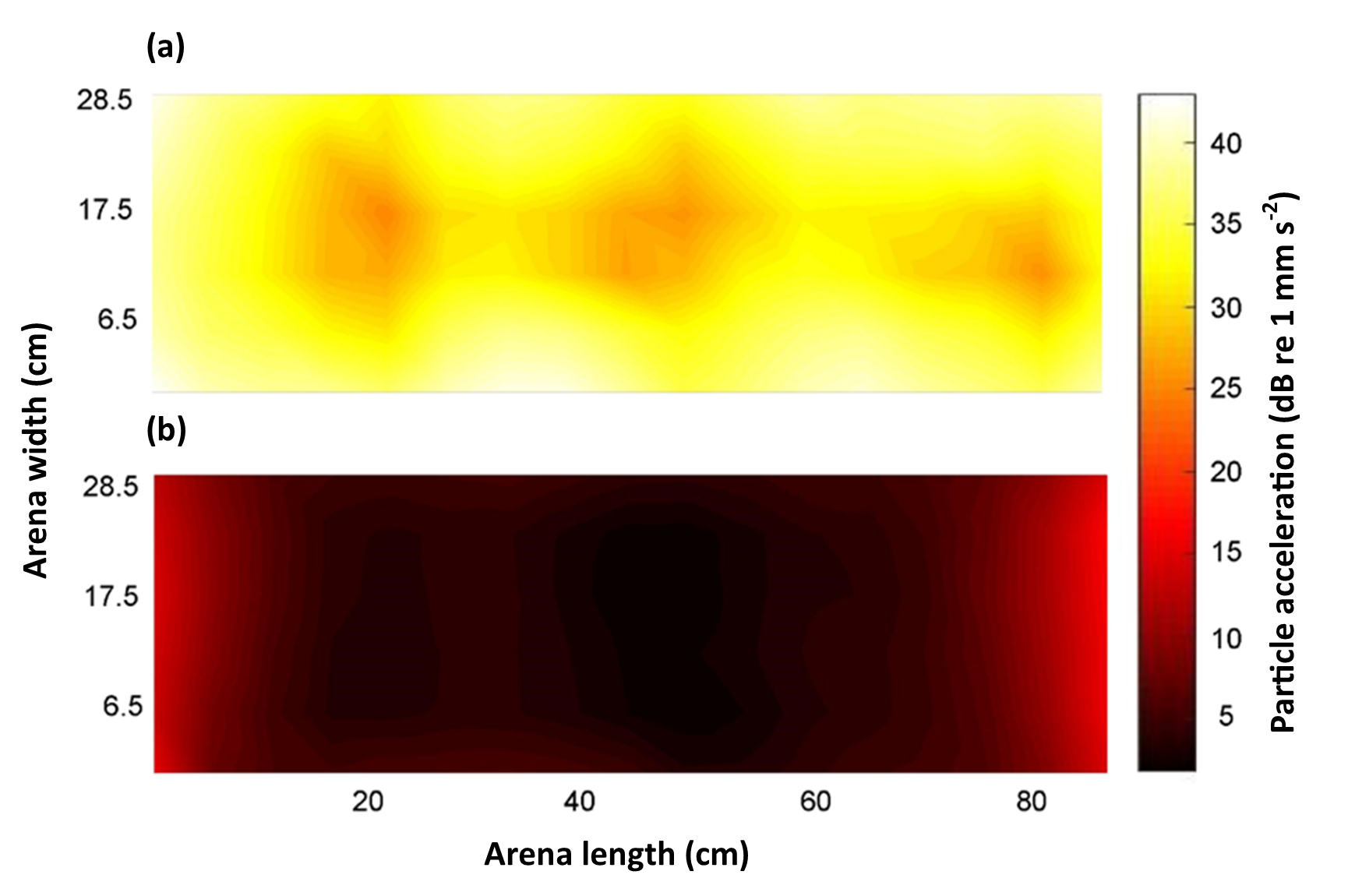

Supplement: S2 Fig — (TIF) [file pone.0327092.s002.tif]
