# Supplementary material for: Masking noise reduces the anti-predator-like response to an acoustic stimulus: Application of Signal Detection Theory to fish behaviour
Source: PLoS One. 2025 Jul 11;20(7):e0327092. doi: 10.1371/journal.pone.0327092 (PMC12250208; doi:10.1371/journal.pone.0327092)
Supplement: S2 Table — Note: Grey shading indicates that a trial (01−10) deviated from the group “normative fit” (regression line equation: y = 0.217 + 3.84 x 10−5 x; ± s.e. = ± 2.04 x 10^−5^; CI [−1.70 x 10^−6^; 7.84 x 10^−5^]) and was classed as a “false alarm” (incorrect response for control) or “hit” (correct response for treatments). (DOCX) [file pone.0327092.s004.docx]

**S2 Table:** False alarm (FAR) and hit rates (HR) for trial groups exposed to 170 Hz tonal stimuli under ambient noise treatments determined through the calculation of generalised least squares regression models. Note: Grey shading indicates that a trial (01-10) deviated from the group “normative fit” (regression line equation: y = 0.217 + 3.84 x 10^-5^ x; ± s.e. = ± 2.04 x 10^-5^; CI [-1.70 x 10^-6^; 7.84 x 10^-5^]) and was classed as a “false alarm” (incorrect response for control) or “hit” (correct response for treatments).

| AMB-C *(FAR = 0.0)* | 01 | 02 | 03 | 04 | Trial no° 05 06 | | 07 | 08 | 09 | 10 |
| --- | --- | --- | --- | --- | --- | --- | --- | --- | --- | --- |
| Lower CI | -1.07 x 10^-5^ | -7.01 x 10^-5^ | -7.17 x 10^-5^ | -1.10 x 10^-4^ | -2.02 x 10^-4^ | -9.46 x 10^-6^ | 1.74 x 10^-5^ | -1.13 x 10^-4^ | 3.22 x 10^-5^ | -1.65 x 10^-4^ |
| Upper CI | 1.53 x 10^-4^ | 1.32 x10^-4^ | 2.03 x 10^-4^ | 1.16 x 10^-4^ | 2.69 x 10^-5^ | 2.23 x10^-4^ | 2.12 x 10^-4^ | 1.13 x 10^-4^ | 2.16 x 10^-4^ | 7.78 x 10^-5^ |
| AMB-LOW *(HR = 0.4)* | **01** | **02** | **03** | **04** | **05** | **06** | **07** | **08** | **09** | **10** |
| Lower CI | -1.25 x 10^-4^ | -2.21 x 10^-4^ | -5.33 x 10^-5^ | -1.76 x 10^-4^ | -2.00 x 10^-4^ | -1.50 x 10^-4^ | -2.77 x 10^-4^ | 3.20 x 10^-4^ | -8.33 x 10^-5^ | 5.48 x 10^-5^ |
| Upper CI | 4.79 x 10^-5^ | -2.60 x 10^-5^ | 6.04 x  10^-5^ | 3.09 x 10^-5^ | 8.21 x 10^-5^ | -5.10 x 10^-5^ | -1.24 x 10^-4^ | 5.42 x 10^-4^ | 1.35 x 10^-4^ | 2.92 x 10^-4^ |
| AMB-INT *(HR = 0.5)* | **01** | **02** | **03** | **04** | **05** | **06** | **07** | **08** | **09** | **10** |
| Lower CI | -5.53 x 10^-5^ | -1.52 x 10^-4^ | -4.17 x 10^-5^ | -1.41 x 10^-4^ | 5.38 x 10^-5^ | -2.12 x 10^-4^ | 1.87 x 10^-4^ | -2.30 x 10^-4^ | -1.16 x 10^-4^ | -9.24 x 10^-5^ |
| Upper CI | 1.60 x 10^-4^ | -1.75 x 10^-5^ | 1.52 x 10^-4^ | 1.21 x 10^-5^ | 2.95 x 10^-4^ | -2.76 x 10^-6^ | 4.22 x 10^-4^ | -6.83 x 10^-5^ | -4.35 x 10^-5^ | 3.37 x 10^-6^ |
| AMB-HIGH *(HR = 0.8)* | **01** | **02** | **03** | **04** | **05** | **06** | **07** | **08** | **09** | **10** |
| Lower CI | -3.95 x 10^-4^ | -1.50 x 10^-4^ | -2.43 x 10^-4^ | -9.24 x 10^-5^ | -3.88 x 10^-4^ | -1.91 x 10^-4^ | 1.67 x 10^-4^ | -2.43 x 10^-4^ | -2.02 x 10^-4^ | 1.75 x 10^-4^ |
| Upper CI | -6.45 x 10^-5^ | 2.58 x 10^-5^ | -3.25 x 10^-5^ | 5.74 x 10^-5^ | -8.11 x 10^-5^ | -5.22 x 10^-5^ | 3.20 x 10^-4^ | -5.77 x 10^-5^ | -6.45 x10^-5^ | 4.93 x 10^-4^ |
